# Impacts of Environmental Regulation on the Green Transformation and Upgrading of Manufacturing Enterprises

**DOI:** 10.3390/ijerph17207680

**Published:** 2020-10-21

**Authors:** Liang Shen, Runjie Fan, Yuyan Wang, Zhaoqing Yu, Rongyun Tang

**Affiliations:** 1 School of Public Finance and Taxation, Shandong University of Finance and Economics, Jinan 250014, China; 20067433@sdufe.edu.cn (L.S.); frj960312@mail.sdufe.edu.cn (R.F.); 2 School of Management Science and Engineering, Shandong University of Finance and Economics, Jinan 250014, China; 191106006@mail.sdufe.edu.cn; 3 Department of Industrial and System Engineering, University of Tennessee, Knoxville, TN 37996, USA; rtang7@vols.utk.edu

**Keywords:** environmental regulation, manufacturing enterprises, green transformation and upgrading, green technology innovation level

## Abstract

Since environmental problems are becoming increasingly prominent, macro policies and social development have placed higher requirements on manufacturing enterprises to promote green transformation and upgrading (GTU) in China. Considering that different manufacturing enterprises choose different green technology innovation levels for GTU under environmental regulation, a game model between manufacturing enterprises and the government is constructed. The relationship between the green technology innovation level (GTIL) and the environmental regulation intensity is analyzed. Through numerical examples, the influences of environmental regulation and consumer preference on system decisions are further examined. Moreover, an econometric model is constructed to explore the influence that the environmental regulation exerts on the GTIL using panel data from the Chinese manufacturing industry. Our results show that the increase in environmental regulation intensity contributes to improving GTIL and promoting the GTU of manufacturing enterprises. Furthermore, as the environmental regulation is enhanced, the sales price decreases, benefiting consumers. Consumers’ preference for high-GTIL products is conducive to GTU under environmental regulation. Empirical analysis shows that there is a U-shaped relationship between environmental regulation and the GTIL. Only when the intensity reaches a threshold can the environmental regulation be beneficial to improve the GTIL and promote the GTU of Chinese manufacturing enterprises.

## 1. Introduction

Rapid economic development has caused tremendous pressure on the environment. The 2018 Environmental Performance Index shows that as leaders in emerging economies, China and India are ranked 120th and 177th, respectively, in terms of environmental quality. In China, the total manufacturing energy consumption of the manufacturing industry accounted for about 54.7% of the total annual energy consumption in 2017, according to the statistics from the National Bureau of Statistics of China. Chinese governments promised the world and the public that they have been committed to upgrading from a manufacturer of quantity to one of quality, so the green transformation and upgrading (hereinafter referred to as GTU) of manufacturing enterprises are crucial. GTU refers to the fact that manufacturing enterprises should pay attention to the upgrading of production technology, research, improve and innovate existing production technology, and strive to ensure the efficient, clean, low-carbon, and sustainable development of the production process, and finally realize green manufacturing. The Chinese government issued Made in China 2025 in 2015, which called for accelerating the GTU of manufacturing enterprises and improving the efficiency of manufacturing resource utilization. Governments in other countries have also introduced many policies to promote GTU.

The GTU of manufacturing enterprises is closely related to the green technology innovation [1,2,3]. It is generally believed that advanced green innovation technologies can help companies increase productivity, enhance core competitiveness, and promote GTU [4,5,6,7]. However, Genc and De Giovanni [8] pointed out that green technology innovation may have two different effects for green production, namely compliance costs and innovation offsets. Therefore, the impact of green technology innovation on the GTU is uncertain. When green technology innovation increases the production cost, manufacturing enterprises pay more, resulting in lower profits, which is not conducive to the GTU, when green technology innovation reduces the production cost, manufacturing enterprises have more disposable profits to spend on innovation, which is beneficial for the GTU. Bi et al., [9] studied these two scenarios in which technological innovation may increase or decrease production costs. They analyzed the impact of technological innovation cost parameter on pricing strategy, but the technological innovation was considered as an exogenous variable and their study lacked the internal mechanism of cost parameter on technological innovation level. Considering government subsidies are limited by budget, they also found that the influence coefficient of innovative technologies on costs can affect which companies the government chooses to subsidize. In addition to the impact on production costs, GTU also generates costs due to the purchase of production equipment, daily maintenance, staff salary, and other reasons. These can be called the costs of GTU. Therefore, in this paper, the cost of GTU is incorporated as a variable cost that the manufacturing enterprise needs to consider.

The cost of GTU caused by green technological innovation, such as purchasing new production equipment and introducing talent, can increase the cost pressure of manufacturing enterprises. Therefore, GTU has encountered great resistance, and manufacturing enterprises lack the initiative. As the main body of supervision over the GTU of manufacturing enterprises, the government is supposed to adopt certain incentive measures to promote the GTU. The government has developed many measures of environmental regulation for GTU [10,11,12], including sewage charges, subsidies, carbon rights trading system, pollution control deposits, and tax relief.

It is generally believed that strict environmental regulation is conducive to environmentally friendly manufacturing [13,14]. Porter and Der Linde [15] put forward the Porter hypothesis, believing that enterprises can be motivated to innovate and improve productivity due to pressure from environmental regulation. Thus, enhanced productivity can offset the costs of environmental protection and contribute to the enterprises’ profitability. Many scholars have verified the Porter hypothesis. For example, through the study on the manufacturing industry in Taiwan, Yang et al., [16] showed that environmental regulation can increase research and development costs, thereby improving enterprise productivity and competitiveness. Cao et al., [17] researched the direct effect and the moderating effect of environmental regulation on technological innovation, which turns out to be a U-shaped relationship. Krass et al., [18] found that a certain range of taxation can promote enterprises to improve green production technology, but excessive taxation is counterproductive. Moreover, when the government considers environmental benefits, taxation can promote the improvement of green technology innovation. Chen et al., [19] analyzed how two government subsidies (product subsidy or innovation technology subsidy) influence enterprises’ profits and innovation technology levels, suggesting that the government should not use both subsidies at the same time.

However, some researchers also point out that the Porter hypothesis is not always supported. The strict environmental regulation is not always conducive to environmentally friendly manufacturing. Blanco et al., [20] found out that voluntary agreements and voluntary environmental declarations for sustainable products were more efficient than stringent legislations in papermaking. The inability of environmental regulation to promote environmentally friendly manufacturing may be due to the environmental awareness of consumers and enterprises [21]. Zhao and Sun [22] showed that environmental regulation positively affects enterprise innovation but negatively affects enterprise competitiveness in a slight manner. As the government offsets the cost of enterprises through subsidies or tax reduction and exemption, the Porter hypothesis cannot be supported. The experimental research on the green economic development of South Korea and policy influence by Sonnenschein et al., [23] indicated that carbon emissions had increased during the initial period of environmental regulation, and the policies did not work. Yuan et al., [24] also found that environmental regulation is not enough to improve the ecological efficiency, and the production efficiency of the industry determines how environmental regulation influences technological innovation and ecological efficiency.

To sum up, there are extensive studies on environmental regulation, but most are empirical studies that build econometric models to examine the impact of GTU on the manufacturing industry [16,25]. There is scant literature simultaneously using the game model to conduct theoretical analysis and the econometric model to verify conclusions through empirical analysis. In order to analyze the influence mechanism of government environmental regulation, the green technology innovation level (hereinafter referred to as GTIL) is introduced to reflect the GTU of manufacturing companies. The higher the GTIL, the better the GTU of manufacturing enterprises. The innovation of this paper lies in:

Firstly, inspired by the model established to investigate microeconomic impacts of regulation policies in previous literature [11,26], a game model consisting of government and manufacturing enterprises is established to analyze how environmental regulation influences GTU.

Secondly, this paper considers that environmental regulation taken by governments to supervise GTU has different impacts on manufacturing enterprises, that is, environmental regulation rewards the enterprise with a high GTIL, but punishes the enterprise with a low GTIL.

Thirdly, unlike the existing literature that only studies the innovation technology selection of a single enterprise under government subsidies [18], two manufacturing enterprises with different levels of green technology innovation are introduced in this paper.

Finally, an econometric model between environmental regulation and green total factor productivity is established, to perfect the analysis of impacts of environmental regulation on GTU by taking multiple influence factors into account.

This paper aims to explore the relationship between governments’ environmental regulation and the GTU of manufacturing enterprises through a game model and an empirical model. The game model between government and manufacturing enterprises explores the influence mechanism of environmental regulation on the GTU of manufacturing enterprises from the micro level, and the empirical analysis using Chinese manufacturing industry data analyzes the impact of environmental regulation on the GTU of manufacturing enterprises from the macro level. This paper attempts to address three problems: (1) Due to the different conditions of each enterprise, the influences of environmental regulation are different. How is the GTU of manufacturing enterprises with different GTIL influenced? (2) Under environmental regulation, how do the environmental regulation intensity and consumers’ preference affect the sales price, the level of green technology innovation, and other decisions? (3) Is the conclusion of the game model different from that of empirical analysis? Why?

The framework of this paper is as follows. Section 1 introduces the research background and reviews related literature. Section 2 studies the influence mechanism by constructing a game model and analyzing optimal decisions under environmental regulation. Section 3 conducts a numerical analysis of the game model. Section 4 uses empirical analysis to explore how environmental regulation influences GTU. Conclusions and management implications are present in Section 5.

## 2. The Influence Mechanism of Environmental Regulation on GTU of Manufacturing Enterprises

### 2.1. Model Assumptions and Explanations

Under the government’s environmental regulation, what GTIL an enterprise chooses for GTU is affected by many factors, including its situation, competitive environment, and regulatory measures. Assume that there are two types of manufacturing enterprises in the market, who adopt two different levels of green technology innovation to promote GTU. To facilitate problem analysis, the research problem is transformed into the following model using a game theoretical approach to solve.

It is assumed that two enterprises are representing the two types of manufacturing enterprises in the market: enterprises one and two. Enterprise one, who has low economic strength and poor innovation ability, can only promote GTU through a lower GTIL, which is denoted by $h_{1}(h_{1}>0)$. However, enterprise two is just the opposite, who has strong economic strength and market competitiveness, so a higher GTIL is denoted by $h_{2}(h_{2}>h_{1}>0)$ for enterprise two. $h_{i}\left(i=1,2\right)$ denotes GTIL, and the higher the GTIL of an enterprise, the stronger the ability of GTU.

In order to promote GTU, the government sets certain regulation conditions. Assume that there is a lower limit of the level of green technology innovation (hereinafter referred to as lower limit) to conduct environmental regulation, that is, the lower limit for GTU is $h_{0}(h_{0}>0)$, and it is assumed that $h_{2}>h_{0}>h_{1}$. The intensity of environmental regulation is δ(δ>0). Enterprise two gets rewards with the amount of $\delta \left(h_{2}-h_{0}\right)$ as the GTIL reaches $h_{0}$, but enterprise one gets penalties with the amount of $\delta \left(h_{0}-h_{1}\right)$ as the GTIL does not reach $h_{0}$.

For enterprises one and two, to meet the lower limit $h_{0}$ under environmental regulation, it is necessary to invest resources to improve their GTIL, including physical capital and human input. According to Raz et al., [27], Bi et al., [9] and Yenipazarli [5], it is assumed that the GTU cost of enterprise i(i=1,2) is $c_{i}=k_{i}h_{i}{}^{2},\;i=1,2$, where, $k_{i}$ denotes the cost coefficient of GTU. Suppose $v_{i}$ denotes the unit production cost, $p_{i}$ denotes the sales price of products, the market demand is $q_{i}$, and the profit of enterprise is $\pi_{i}$.

The consumers’ preference model of Moorthy [28] is used to analyze consumers’ behavior. Consumers’ preference for products is only affected by sales prices, without considering consumers’ environmental awareness, that is, demand for products is not affected by GTIL. Assuming that the market size is standardized to one, the utility of consumers buying high-GTIL products is u, where u follows a uniform distribution on [0,1] and its distribution function is F(u)=u. The coefficient of consumers’ preference for low-GTIL products is l(0<l<1), then consumers’ utility is lu.

Consumers make choices by comparing the utility surpluses of different products in the market. Products of the two manufacturing enterprises are in a competitive relationship, and consumers choose to buy products with high utility surpluses. When $p_{2}>\left(p_{2}-p_{1}\right)/\left(1-l\right)$, that is, $lp_{2}<p_{1}$, $l-p_{2}\geq 0$ and $l-p_{2}>lu-p_{1}$, consumers only buy high-GTIL products, and low-GTIL products will be pushed out of the market. When $u-p_{2}=lu-p_{1}$, consumers can get the same utility when they buy two kinds of products. Therefore, the utility of consumers buying low-GTIL products is

(1) $$U_{1}=\int_{0}^{\frac{p_{2}-p_{1}}{1-l}}\left(lu-p_{1}\right)dF\left(u\right)=\frac{\left(p_{2}-p_{1}\right)\left[lp_{2}-\left(2-l\right)p_{1}\right]}{2{\left(1-l\right)}^{2}}$$

The utility of consumers buying high-GTIL products is

(2) $$U_{2}=\int_{\frac{p_{2}-p_{1}}{1-l}}^{1}\left(u-p_{2}\right)dF\left(u\right)=\left(1-\frac{p_{2}-p_{1}}{1-l}\right)\left[\frac{1}{2}+\frac{p_{2}-p_{1}}{2\left(1-l\right)}-p_{2}\right]$$

The market demand for high-GTIL products is

(3) $$q_{2}=\int_{\frac{p_{2}-p_{1}}{1-l}}^{1}dF\left(u\right)=1-\frac{p_{2}-p_{1}}{1-l}$$

The market demand for low-GTIL products is

(4) $$q_{1}=\int_{0}^{\frac{p_{2}-p_{1}}{1-l}}dF\left(u\right)=\frac{p_{2}-p_{1}}{1-l}$$

The profits of enterprises and social benefit are

(5) $$\pi_{1}=\left[p_{1}-v_{1}-\delta \left(h_{0}-h_{1}\right)\right]q_{1}-k_{1}h_{1}^{2}$$

(6) $$\pi_{2}=\left[p_{2}-v_{2}+\delta \left(h_{2}-h_{0}\right)\right]q_{2}-k_{2}h_{2}^{2}$$

(7) $$\pi =\pi_{1}+\pi_{2}+U_{1}+U_{2}$$

### 2.2. Solution and Analysis

This subsection constructs a Stackelberg game with the government as the leading player and enterprises as the subordinate. Since the government aims to maximize social benefit, and enterprises aim to maximize their profits, the decision sequence is: the government first determines environmental regulation intensity δ given enterprise development and environmental protection; then based on δ, two enterprises determine their respective levels of green technology innovation, $h_{i},\;i=1,2$; finally, two enterprises determine the sales price of products $p_{i}$ based on their GTU. The solution can be derived by backward induction.

According to $\frac{\partial \pi_{1}}{\partial p_{1}}=0$ and $\frac{\partial \pi_{2}}{\partial p_{2}}=0$, the response functions of $p_{1}$ and $p_{2}$ with respect to $h_{1}$ and $h_{2}$ are

(8) $$p_{1}=\frac{1}{3}\left(1-l+3\delta h_{0}-2\delta h_{1}-\delta h_{2}+2v_{1}+v_{1}\right)$$

(9) $$p_{2}=\frac{1}{3}\left(2-2l+3\delta h_{0}-\delta h_{1}-2\delta h_{2}+v_{1}+2v_{1}\right)$$

Substitute Equations (8) and (9) into Equations (5) and (6), functions of $\pi_{1}$ and $\pi_{2}$ with respect to $h_{1}$ and $h_{2}$ can be derived. According to $\frac{\partial \pi_{1}}{\partial h_{1}}=0$ and $\frac{\partial \pi_{2}}{\partial h_{2}}=0$, response functions of $h_{1}^{*}$ and $h_{2}^{*}$ with respect to δ are

(10) $$h_{1}^{*}=\frac{\delta^{3}+3\delta k_{2}\left[\left(v_{1}-v_{2}\right)-\left(1-l\right)\right]}{3\left[\delta^{2}\left(k_{1}+k_{2}\right)-9k_{1}k_{2}\left(1-l\right)\right]}$$

(11) $$h_{2}^{*}=\frac{\delta^{3}+3\delta k_{1}\left[\left(v_{2}-v_{1}\right)-2\left(1-l\right)\right]}{3\left[\delta^{2}\left(k_{1}+k_{2}\right)-9k_{1}k_{2}\left(1-l\right)\right]}$$

Substitute Equations (10) and (11) into Equations (8) and (9), response functions of $p_{1}^{*}$ and $p_{2}^{*}$ with respect to δ are

(12) $$p_{1}^{*}=\frac{-\delta^{4}+3\left(1-l\right)\left[\delta^{2}\left(k_{1}+k_{2}\right)-3k_{1}k_{2}\left(1-l+3\delta h_{0}\right)\right]+3\left[\delta^{2}\left(k_{1}v_{1}+k_{2}v_{2}\right)+\delta^{3}h_{0}\left(k_{1}+k_{2}\right)-3k_{1}k_{1}\left(1-l\right)\left(2v_{1}+v_{2}\right)\right]}{3\left[\delta^{2}\left(k_{1}+k_{2}\right)-9k_{1}k_{2}\left(1-l\right)\right]}$$

(13) $$p_{2}^{*}=\frac{-\delta^{4}+3\left(1-l\right)\left[\delta^{2}\left(2k_{1}+k_{2}\right)-3k_{1}k_{2}\left(2-2l+3\delta h_{0}\right)\right]+3\left[\delta^{2}\left(k_{1}v_{1}+k_{2}v_{2}\right)+\delta^{3}h_{0}\left(k_{1}+k_{2}\right)-3k_{1}k_{1}\left(1-l\right)\left(v_{1}+2v_{2}\right)\right]}{3\left[\delta^{2}\left(k_{1}+k_{2}\right)-9k_{1}k_{2}\left(1-l\right)\right]}$$

It can be further obtained that the market demand of the two enterprises is

(14) $$q_{1}^{*}=\frac{k_{1}\left(\delta^{2}+3k_{2}\left(-1+l+v_{1}-v_{2}\right)\right)}{\delta^{2}k_{2}+k_{1}\left(\delta^{2}+9\left(-1+l\right)k_{2}\right)}$$

(15) $$q_{2}^{*}=\frac{k_{2}\left(\delta^{2}+3k_{1}\left(-2+2l-v_{1}+v_{2}\right)\right)}{\delta^{2}k_{2}+k_{1}\left(\delta^{2}+9\left(-1+l\right)k_{2}\right)}$$

According to the above results, $\pi_{1}^{*}$ and $\pi_{2}^{*}$ can be obtained as

(16) $$\pi_{1}^{*}=\frac{k_{1}\left[9k_{1}\left(1-l\right)-\delta^{2}\right]{\left\{\delta^{2}-3k_{2}\left[\left(1-l\right)+\left(v_{2}-v_{1}\right)\right]\right\}}^{2}}{9{\left[\delta^{2}\left(k_{1}+k_{2}\right)-9k_{1}k_{2}\left(1-l\right)\right]}^{2}}$$

(17) $$\pi_{2}^{*}=\frac{k_{2}\left[9k_{2}\left(1-l\right)-\delta^{2}\right]{\left\{\delta^{2}-3k_{2}\left[2\left(1-l\right)+\left(v_{1}-v_{2}\right)\right]\right\}}^{2}}{9{\left[\delta^{2}\left(k_{1}+k_{2}\right)-9k_{1}k_{2}\left(1-l\right)\right]}^{2}}$$

Substitute $p_{1}^{*}$, $p_{2}^{*}$, $h_{1}^{*}$, $h_{2}^{*}$ into $U_{1}$ and $U_{2}$, the utility of buying products from enterprise one is $U_{1}^{*}$ and the utility of buying products from enterprise two is $U_{2}^{*}$. Therefore, social benefit is

(18) $$\pi^{*}=\pi_{1}^{*}+\pi_{2}^{*}+U_{1}^{*}+U_{2}^{*}$$

It is generally believed that if the lower limit is not met, the penalties will result in a decline in profits, if the lower limit is met, the rewards will increase profits. However, Equations (16) and (17) show that the lower limit set by the government does not affect the profits of enterprises. This is because the increase in the lower limit has driven the increase in sales prices, and consumers have to pay more at a high sales price. Therefore, the government should consider consumers’ concerns about sales prices when setting this lower limit. This shows that the lower limit of GTIL does not have a substantial impact on enterprises’ profits, and the GTU of manufacturing enterprises cannot be effectively regulated by setting this lower limit. The government needs to set a reasonable environmental regulation intensity to affect the GTU of manufacturing enterprises.

When the government does not set environmental regulation (that is, δ=0), it can be calculated according to the profit functions of enterprises one and two (that is, Equations (5) and (6)) that the optimal GTIL is zero when enterprises make decisions intending to maximize profits. This shows that if the government does not take regulatory action, enterprises are reluctant to participate in GTU, and will not actively take measures to undertake responsibility for environmental protection and pollution control. Therefore, for environmental benefits and sustainable development, the government needs to motivate enterprises to promote GTU and achieve sustainable development. The environmental regulation intensity cannot be zero, and it needs to be set according to factors such as economic development and the technological level of manufacturing enterprises.

## 3. Numerical Analysis

**Figure 1:**
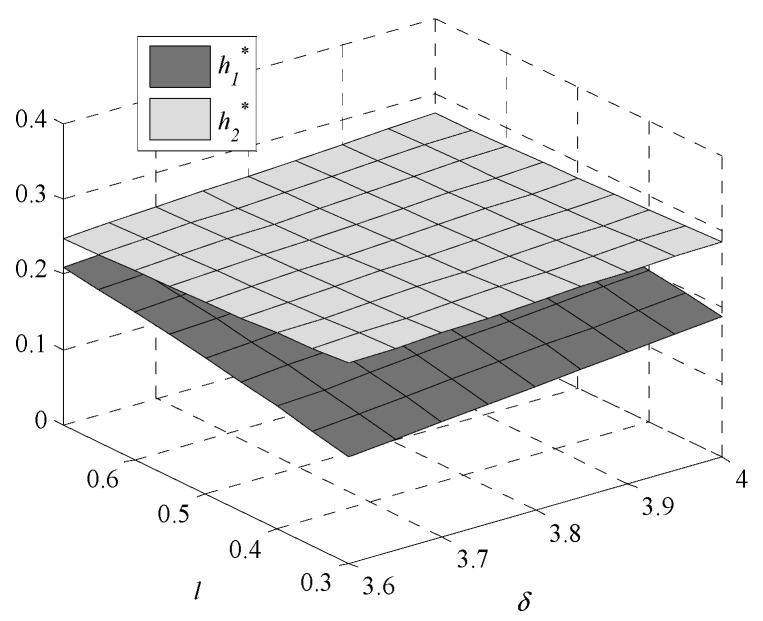
Changes in green technology innovation level (GTIL).

**Figure 2:**
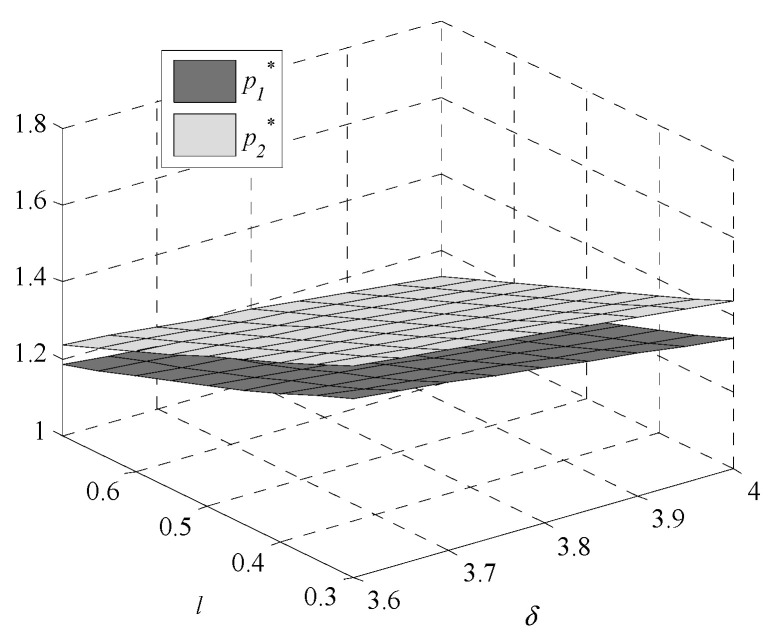
Changes in sales price.

**Figure 3:**
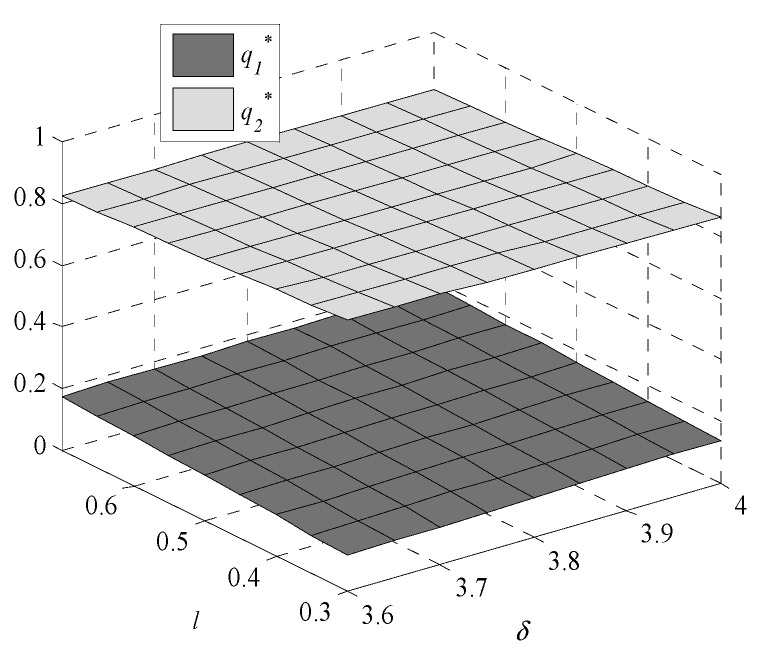
Changes in market demand.

**Figure 4:**
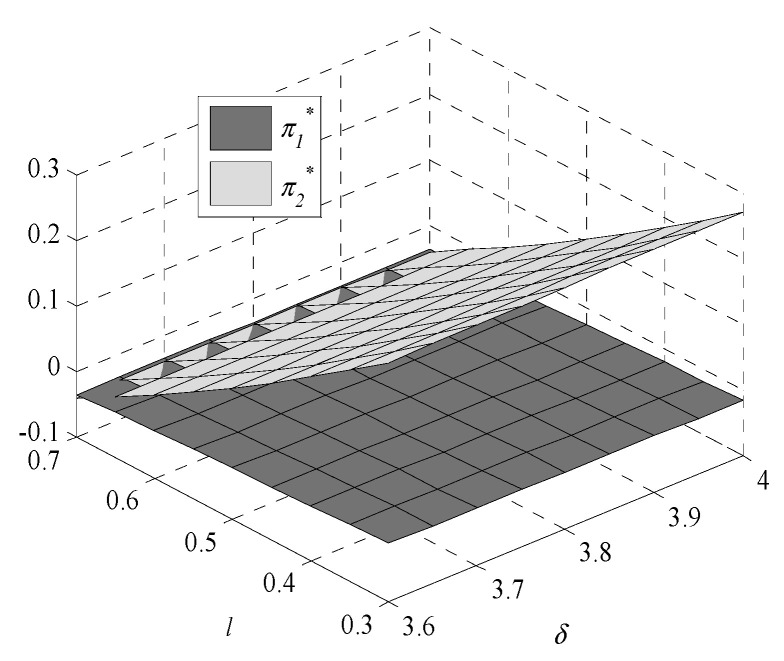
Changes in profit of enterprises.

**Figure 5:**
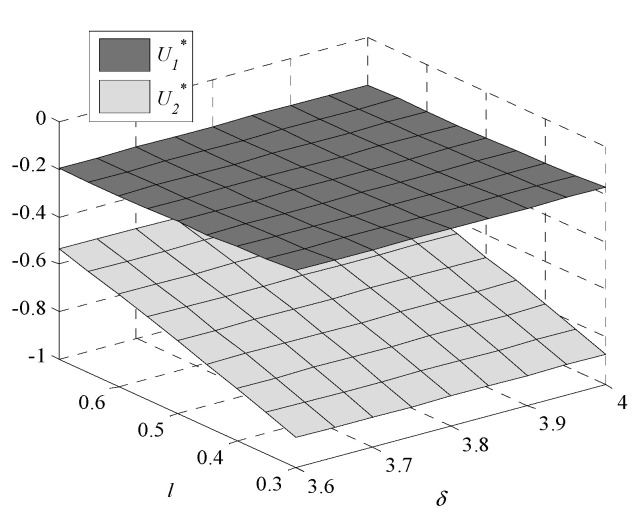
Changes in the utility of the consumers.

**Figure 6:**
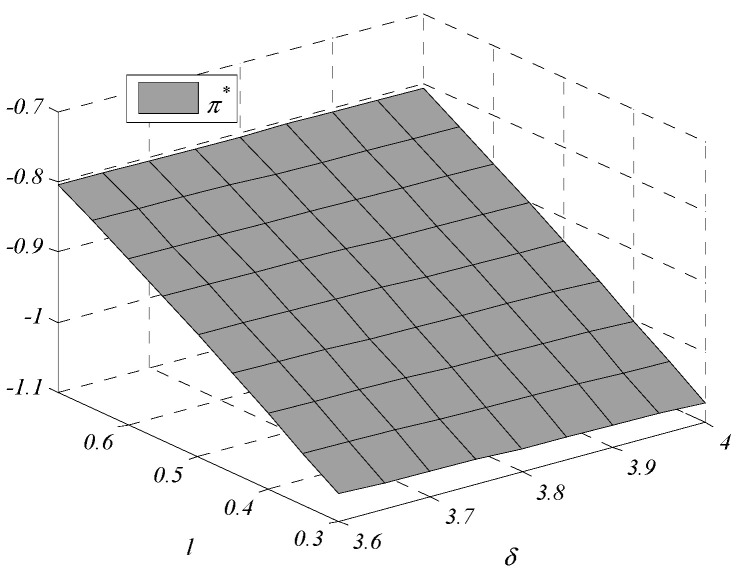
Changes in social benefit.

Given the complexity of expressions, the influences of environmental regulation intensity and consumers’ preference on decisions are analyzed by numerical examples. In this paper, following Bi et al., [9], Krass et al., [18], Gao et al., [29] and Xu et al., [30], we generalize the model and parameters’ values in the game model to meet the assumptions and explanations of Section 2.1. Assume values of parameters are $k_{1}=1$, $\;k_{2}=4$, $\;v_{1}=1.005$, $v_{2}=1$, and $h_{0}=0.245$. The independent variables are δ and l, the changes in decisions with δ∈[3.6,4] and l∈[0.3,0.7] are shown in Figure 1, Figure 2, Figure 3, Figure 4, Figure 5 and Figure 6.

### 3.1. Impact of Environmental Regulation Intensity

Figure 1 shows that the GTIL increases with environmental regulation intensity. For enterprise one, which fails to meet the lower limit, the increased environmental regulation intensity means that the penalties are increased. In order to reduce penalties, enterprise one has to improve the GTIL to meet the lower limit set by the government. However, for enterprise two, which meets the lower limit, the increased intensity of environmental regulations means increased rewards, further promoting enterprise two to improve the GTIL. Therefore, whether environmental regulation is used as a penalty or as a reward, it can promote and incentivize enterprises’ GTU. Additionally, compared with the changing trend of $h_{2}^{*}$ with δ, the changing trend of $h_{1}^{*}$ is more obvious, which indicates that it is more effective when environmental regulation is used as a penalty than a reward.

As environmental regulation intensity increases, the sales price decreases. Enterprise two, which adopts a higher GTIL, makes appropriate concessions to consumers by reducing the sales price to increase market demand and enhance market competitiveness. This forces enterprise one to reduce the sales price to attract consumers.

The profits of enterprise one and enterprise two also decrease with the increase in environmental regulation intensity. This is because as GTIL improves, the cost of GTU increases, the profits of both enterprises decrease. After enterprise participates in GTU, due to the long cycle of green technology innovation, the profits are meager or maybe a loss. Therefore, participation in GTU lacks internal motivation, and it is often a passive choice to adopt green technology innovation. In most cases, the profit of enterprise two is higher than the profit of enterprise one. Only when consumers have a strong preference for low-GTIL products and weak environmental regulation, can the profit of enterprise one be higher than the profit of enterprise two. Therefore, the government needs to regulate GTU, which also indirectly reflects the important role of environmental regulation.

Social benefit also increases with the environmental regulation intensity: when the intensity increases, consumers can buy high-GTIL products at lower sales prices, and consumers’ utility increases. The increase in social benefit is mainly due to the increase in the consumers’ utility.

### 3.2. Impact of Consumers’ Preference for Products

As consumers’ preference for low-GTIL products decreases (preference for high-GTIL products increases), the GTIL of enterprise one decreases but the GTIL of enterprise two increases. This is because when consumers prefer the products with high-GTIL, enterprise two will be motivated to promote GTU. This indicates that the consumers’ preference for high-GTIL products can help enterprise two to actively carry out GTU. However, with the increasing consumers’ preference for high-GTIL products, the low-GTIL products produced by enterprise one are no longer favored by consumers, and enterprise one gradually loses market share. In this case, enterprise one tends to reduce the GTIL to reduce the cost of GTU.

When consumers’ preference for high-GTIL products increases, the sales prices of both enterprises increase. With the increasing preference for high-GTIL products, the GTIL of enterprise two is becoming higher, leading to an increase in the cost of enterprise two for GTU. Therefore, enterprise two has to improve its sales price to ensure the profit. Enterprise one is forced to raise the sales price to ensure the profit due to the decrease in demand for low-GTIL products.

It is straightforward that as consumers’ preference for products with high-GTIL increases, the products demand of enterprise one decreases but the products demand of enterprise two increases. When the preference for high-GTIL increases, products of enterprise two are more popular than products of enterprise one.

The profits of enterprise one and enterprise two increase with the enhancement of consumers’ preference for high-GTIL products, showing that consumers’ preference for high-GTIL products is beneficial to the long-term development of enterprises. Therefore, in addition to regulating the GTU of enterprises, the government can also increase green publicity to raise consumers’ awareness of green consumption and promote enterprise development.

## 4. Empirical Analysis

In actual operation, GTU can be affected by many factors, resulting in a more complicated relationship between environmental regulation and GTIL. Thus, empirical analysis is carried out in this section to explore the relationship.

### 4.1. Model Construction and Variable Description

In the econometric model, control variables and explained variable are logarithmized to reduce the effect of heteroscedasticity, and the quadratic term of environmental regulation is introduced to research the non-linear function relationship. The econometric model in this paper studies the positive and negative correlations and does not focus on the size of the correlation coefficient.

(19) $$lnGTIL_{it}=\alpha +\beta_{1}GER_{y-it}+\beta_{2}GER_{y-it}^{2}+\beta_{3}lnCI_{it}+\beta_{4}lnLI_{it}+\beta_{5}lnAGDP_{it}+\beta_{6}lnISL_{it}+\mu_{i}+\lambda_{t}+\delta_{it}$$

In Equation (19), α represents a constant term, i and t, respectively, represent region and year, $\beta_{i}$ represents a parameter to be estimated, $\delta_{it}$ represents the random error, $\;\mu_{i}$ and $\lambda_{t}$, respectively, represent individual fixed effects and time fixed effects, and y=1 represents the one-year lag.

(1) Explanatory variable

The explanatory variables include control variables and the independent variable. This paper selects the capital input, labor input, economic development level, and industrial structure level as the control variables. The main inputs in the Cobb–Douglas production function are labor and capital, so capital input and labor input are selected as control variables [31], where capital input (CI) is denoted by fixed assets invested in manufacturing (unit: 100 million yuan), labor input (LI) is measured by employees of private manufacturing enterprises and individual business (unit: 10,000). The GTU can be also affected by the economic development level and industrial structure level [30]. The economic development level is measured by average gross domestic product (AGDP) (unit: yuan/person), and the industrial structure level (ISL) uses the ratio between the added value of the tertiary industry and the added value of the secondary industry.

The independent variable is the environmental regulation (GER), that is, the government’s regulation on GTU. There are many methods to measure this indicator in the literature. With reference to Cao et al., [17] and Liu et al., [32], the ratio of the investment completed in the treatment of industrial pollution to the industrial added value of this year is selected to measure this variable. Since manufacturing belongs to the category of the industry in China and considering the availability of data, industry data are selected for calculation.

(2) Explained variable

Green total factor productivity is used to measure the level of green technology innovation (GTIL), this measurement uses an efficiency measurement model that takes into account undesired output [33]. When calculating the green total factor productivity of manufacturing enterprises, the input variables are fixed assets, the number of employees, and investment completed in the treatment of industrial pollution. Affected by environmental regulation, many manufacturing enterprises have carried out green technology innovation. Given data availability and considering that manufacturing belongs to the category of the industry in China, the industrial added value is selected as expected output. The undesired output is the total industrial wastewater discharge and sulfur dioxide emissions [34]. For data envelopment analysis (DEA), we used DEA-Solver Pro 5.0 to calculate the green total factor productivity.

Based on data availability, panel data from 30 provinces and cities in China from 2004 to 2017 is used for empirical research. The relevant data come from *China Statistical Yearbook* and *China Industrial Statistical Yearbook*. The empirical analysis is conducted using Stata14.

### 4.2. Results Analysis

Before performing regression analysis, Im-Pesaran-Shin (IPS) unit-root test is conducted for explanatory variables, and it is found that labor input and industrial structure level have unit-roots. The test shows that the original value sequence is stable after the first-order difference. The *p* value of F-test using fixed effect is 0.0000, indicating that the null hypothesis (that the pooled regression is accepted) can be strongly rejected. Therefore, the fixed effects regression is more suitable than the pooled regression. The Hausman test shows that the *p* value is 0.0000, indicating that fixed effects regression is more suitable than the random effects regression. Therefore, the fixed effects regression model is employed to examine the effects of environmental regulation on GTIL, controlling year fixed effects and provinces and cities fixed effects (i.e., two-way fixed effects). The regression results are shown in Table 1. The method of gradually adding control variables is used to perform regression to ensure the reliability of regression results. Table 1 shows that as control variables are added (models (1)–(5)), regression coefficients of environmental regulation are significantly negative at 1%, and regression coefficients of the quadratic term are significantly positive at 1%. Meanwhile, considering environmental regulation’s lagging effect [35], the regression results of the one-year lag are conducted, which is model (6) in Table 1.

The regression results show that coefficients of environmental regulation are negative, and coefficients of the quadratic term are positive, which are all statistically significant, so does the coefficients of one-year lag. It is indicated that there is a U-shaped relationship between environmental regulation and GTIL. As environmental regulation intensity increases, GTIL decreases first and then rises. This effect also exists in the analysis of the one-year lag. As control variables are added gradually, the positive and negative coefficients of environmental regulation are stable, which also shows the stability of the relationship and the reliability of the model.

With the addition of control variables, the coefficients of each control variable are statistically significant in model (5). It is shown that coefficients of capital input, labor input, and industrial structure level are negative, while the coefficient of economic development level is positive. When the capital input and labor input are large, it means that the transformation cost of the enterprise is greater, so the impact is negative. The increase in the economic development level will increase the quality requirements for economic development, so the requirements for green total factor productivity will increase and have a positive effect. The increased pressure is helpful for the improvement of the GTU of manufacturing enterprises. The higher the industrial structure level means that the ratio of the tertiary industry to the secondary industry is greater, and the development of the manufacturing is limited. Therefore, the impact is negative.

The aforementioned analysis shows that when environmental regulation intensity is low, the GTIL decreases with the increase in the intensity. However, if the environmental regulation intensity exceeds a certain value, GTIL increases with the intensity. This is inconsistent with the analysis of the game model, which shows that the GTIL is positively related to environmental regulation. This is because the game model discusses the government’s environmental regulation under a single period. A single period is considered to be the mature stage of product life cycle, so decision variables are stable [36,37]. Production resources are assumed to be sufficient under a single period, and resource constraints in the early stage are not considered. In the early stage of environmental regulation, when the intensity is low, enterprises need to pay a certain cost for green technology innovation. In this case, enterprises face constraints such as capital and human, so the environmental regulation plays a negative role, that is, as environmental regulation intensity increases within a certain range, GTIL decreases. However, if the intensity exceeds a threshold, the penalties faced by enterprises with lower GTIL increase sharply, and the rewards for enterprises with higher GTIL increase dramatically. Therefore, the GTIL increases with environmental regulation intensity to avoid penalties for low-GTIL enterprises and obtain rewards for high-GTIL enterprises.

## 5. Conclusions

Given the influence of environmental regulation on GTU, this paper builds models consisting of the government and manufacturing enterprises with different GTIL to analyze how environmental regulation influences the GTU. From the micro-level, a game model of environmental regulation acting on GTU is first constructed to analyze the impacts of environmental regulation on GTIL and the pricing of different types of enterprises. Then, an econometric model investigating how environmental regulation affects green total factor productivity is constructed macroscopically. Conclusions are as follows.

The analysis of the game model reveals that environmental regulation contributes to the GTU of manufacturing enterprises. Through environmental regulation, the government can affect the GTIL of enterprises. Without considering constraints such as consumer awareness and the global economic situation, as environmental regulation intensity increases, GTIL, market demand, and social benefits increase, while sales price decreases and enterprises’ profits decrease. The lower limit does not affect the GTIL and enterprises’ profits but can increase the sales price, so the government needs to consider this relationship when setting the lower limit.

Empirical analysis shows that in the actual operation of manufacturing enterprises, there is a U-shaped relationship between environmental regulation and GTU. The difference between the results of the empirical analysis and the conclusions of the game model analysis is mainly because the conclusions of the game model are discussed in a single cycle, without considering the constraints of the manufacturing enterprise during the production process. From the perspective of empirical analysis, the impact of environmental regulation on GTU is limited by constraints from types of enterprises or product types, such as insufficient capital and labor shortages. The GTU of manufacturing enterprises is affected by many factors. This study shows that the impacts of capital input, labor input, and industrial structure level are significantly negative, and the impact of the economic development level is significantly positive.

The aforementioned conclusions have some managerial enlightenment.

For manufacturing enterprises, with the increase in environmental regulation intensity, the improvement of the GTIL can reduce the sales price, which benefits consumers but reduces the profits of enterprises. This is the price that must be paid for the GTU of the manufacturing industry, and the price to undertake social responsibility. Manufacturing enterprises are supposed to consciously undertake social responsibility and actively transform and upgrade to sustainable green development.

From the perspective of consumers, the increase in preference for high-GTIL products is conducive to improving enterprises’ profits. In addition to regulating the GTU, the government can also promote green publicity to raise consumers’ awareness of green consumption, thus stimulating preference for high-GTIL products.

When setting the lower limit, the government should consider the impact on the sales price to avoid an excessive increase in sales price, which can negatively affect product sales. The design of environmental regulation intensity cannot only rely on theoretical model analysis. Specifically, it is necessary to set a reasonable intensity based on practice, combined with economic development and the specific conditions of enterprises.

Moreover, relevant government departments should also encourage small- and medium-sized enterprises to actively promote GTU. It is necessary to improve the regulatory system for small- and medium-sized manufacturing enterprises, formulate specific indicators, and establish reward–penalty mechanisms to encourage GTU.

This paper studies the impacts of government environmental regulation on the GTU of the manufacturing industry. However, there are still limitations. For example, product demands only considers consumers’ preference for prices, without considering consumers’ environmental awareness and enterprises’ attitudes towards the GTU. This paper can be extended from the following two aspects in future research. On one hand, a concrete example from the industry can be presented alongside the study to make the research problem attractive and present a better reflection of the topic. On the other hand, the GTU is not only affected by the environmental regulation from governments, but also the informal environmental regulation from social groups who can negotiate with manufacturing enterprises to affect the GTU of manufacturing enterprises [38,39]. Thus, the dual environmental regulation also requires further research.

## Figures and Tables

**Table 1:** Regression results of the econometric model.

	(**1**)	(**2**)	(**3**)	(**4**)	(**5**)	(**6**)
GER	−115.577 ***	−115.047 ***	−118.454 ***	−112.991 ***	−105.736 ***	
	(−10.91)	(−10.75)	(−11.21)	(−11.74)	(−11.99)	
$GER^{2}$	3451.456 ***	3429.727 ***	3474.165 ***	3336.962 ***	3109.288 ***	
	(7.42)	(7.31)	(7.53)	(7.95)	(8.10)	
lnCI		0.015	0.034	−0.177 ***	−0.197 ***	−0.219 ***
		(0.37)	(0.86)	(−4.12)	(−5.03)	(−4.58)
lnLI			−0.253 ***	−0.338 ***	−0.382 ***	−0.346 ***
			(−3.72)	(−5.39)	(−6.65)	(−5.21)
lnAGDP				1.216 ***	0.482 ***	0.506 ***
				(8.90)	(3.20)	(2.76)
lnISL					−0.933 ***	−1.043 ***
					(−8.71)	(−7.95)
$GER_{1-}$						−70.978 ***
						(−6.97)
$GER_{1-}^{2}$						2374.371 ***
						(5.43)
_cons	−0.241 ***	−0.329	0.563 *	−9.337 ***	−2.266 *	−2.702 *
	(−4.05)	(−1.36)	(1.66)	(−8.09)	(−1.70)	(−1.66)
*N*	420	420	420	420	420	390
Time fixed effect	Yes	Yes	Yes	Yes	Yes	Yes
Provinces and cities fixed effect	Yes	Yes	Yes	Yes	Yes	Yes
R^2^	0.404	0.404	0.425	0.526	0.607	0.504

Notes: *t* statistics in parentheses; *** *p* < 0.01, * *p* < 0.1.
